# Assessment of heavy metals at mangrove ecosystem, applying multiple approaches using in-situ and remote sensing techniques, Red Sea, Egypt

**DOI:** 10.1007/s11356-023-31625-y

**Published:** 2024-01-04

**Authors:** Asmaa H. Mohammed, Ahmed M. Khalifa, Hagar M. Mohamed, Kareem H. Abd El-Wahid, Mahmoud H. Hanafy

**Affiliations:** 1 Marine Sciences Department, National Authority for Remote Sensing and Space Sciences, Cairo, Egypt; 2 Geology Department, National Authority for Remote Sensing and Space Sciences, Cairo, Egypt; 3 Marine Sciences Department, Science College, Suez Canal University, Ismailia, Egypt

**Keywords:** Coastal health status, Geochemical index, Heavy metals pollution, Mangrove ecosystem, Red Sea

## Abstract

Mangrove areas are considered the most retention zone for heavy metal pollution as it work as an edge that aggregates land and sea sediments. This study aims to examine if the heavy metals’ existence in the mangrove sediment is related to contamination or natural resources. In addition, it gives an interpretation of the origin of these metals along the Egyptian Red Sea coast. Twenty-two samples of mangrove sediments were collected and then, analyzed for metals (Mn, Ni, Cu, Fe, Cd, Ag, and Pb) using inductively coupled plasma mass spectroscopy (ICP-MS). Integration between the in-situ data, contamination indices, and remote sensing and geographical information science (GIS), and multivariate statistical analysis techniques (PCA) were analyzed to assess and clarify the spatial origin of heavy metals in sediment at a regional scale. The average concentration of heavy metals from mangrove sediments were shown to be substantially lower than the referenced value, ranging from moderate to significant except the levels of Ag were very high. The heavy metals concentrations were expected to be naturally origin rather than anthropogenic and that be confirmed by mapping of Red Sea alteration zones spots. These alteration zones are parallel to mangrove sites and rich by several mineralization types including heavy metals that are carried by flooding to the coastline. Remote sensing and GIS techniques successfully contributed to interpreting the pattern of the origin of heavy metals and discharging systems that control the heavy metals concentration along the Red Sea coast.

## Introduction

The coastal zones are significant natural ecosystems as transitional between terrestrial and aquatic areas (Crossland et al. 2005). The Red Sea coastal zones have an important role in the blue economy varying from mining, shipping, fisheries, biodiversity-related tourism, etc. (Cziesielski et al. 2021; Kabil et al. 2022). The mangrove habitats are common and dispersed along the Egyptian Red Sea. Mangroves play a vital ecological role as one of the most productive marine habitats (Friess et al. 2020). The mangrove trees provide shelters and feeding nurseries for both land and marine dwellers. The stands of mangroves offer natural protection from erosion and storms. The mangrove forests have an action in the cycle and are considered one of the most carbon-rich ecosystems (Alongi 2020; Almahasheer et al. 2017).

There are two mangrove species in the Egyptian Red Sea the Avicennia marina (gray mangrove) mainly dispreading along the coast from the north and Rhizophora mucronata (red mangrove) located only at the southern borders (Afefe et al. 2019). The mangrove zones are facing different threats, e.g., oil pollution, overcutting, overgrazing, and denudation. These impacts are causing damage and loss in the areas of these appreciated plants (Alongi 2020; Nunoo and Agyekumhene 2022). The mangrove habitat management needs assessment and incessant monitoring through gathering data from different disciplines such as soil contamination assessment and heavy metals concentrations (Schmitt and Duke 2015; De Alban et al. 2020; Afefe 2021).

The unmanaged development activities particularly industries with interference at the coastal zones have increased impacts on the marine environment. The Red Sea province has an intensive tourism development coastline and an increasing population growth and urban development that pressure on coastal environments (Abdel-Latif et al. 2012). In addition to the mining activity in the Red Sea mountains near the coast, there is one uncompleted industry zone which considers anthropogenic impacts (Mohamed 2005; Analuddin et al. 2023). On the other hand, the marine sediments work as reservoirs that concentrate the contaminants especially heavy metals of different discharges from terrestrial and marine (Tripathi et al. 2014; Tang et al. 2019. Heavy metals are mostly toxic contaminants in the environment resulting from non-biodegradable and bioaccumulation processes (El-Said and Youssef 2013; Tam and Wong 2000; Kumar et al. 2020; Elnaggar et al. 2022). The high levels of heavy metal contamination in mangrove forests have been studied comprehensively (Marchand et al. 2006; Praveena et al. 2008; Al-Mutairi and Yap 2021) reported that heavy metals are discharged into coastal mangrove areas due to mining practices, industrial and port activities. The concentration of metals in the environment is recognizing its toxicity to biota from normal to lethal or sub-lethal (Li and Davis 2008; Ama et al. 2014).

The remote sensing techniques were valuable in mapping environmental conditions and clarifying the resources of impacts especially, for the remote areas of mangroves. Mapping of Wadis routes with ancillary data of geologic components brings images of natural metals distributed on the Red Sea coast. Integration of remote sensing with in-situ data on contamination of heavy metals, where sediment quality assessment tools are respected in identifying sediment quality conditions recognized with the biological effects; geo-accumulation index, enrichment factor, contamination factor, degree of contamination, and pollution load index (Shafe et al. 2013; Kumar and Fulekar 2019).

This research aims to evaluate the environmental condition of sediment contamination with heavy metals affecting mangrove areas and to assess the natural and anthropogenic activities related to heavy metal concentrations in mangrove sites along the Egyptian Red Sea coast.

## Material and methods

### Study area

**Fig. 1:**
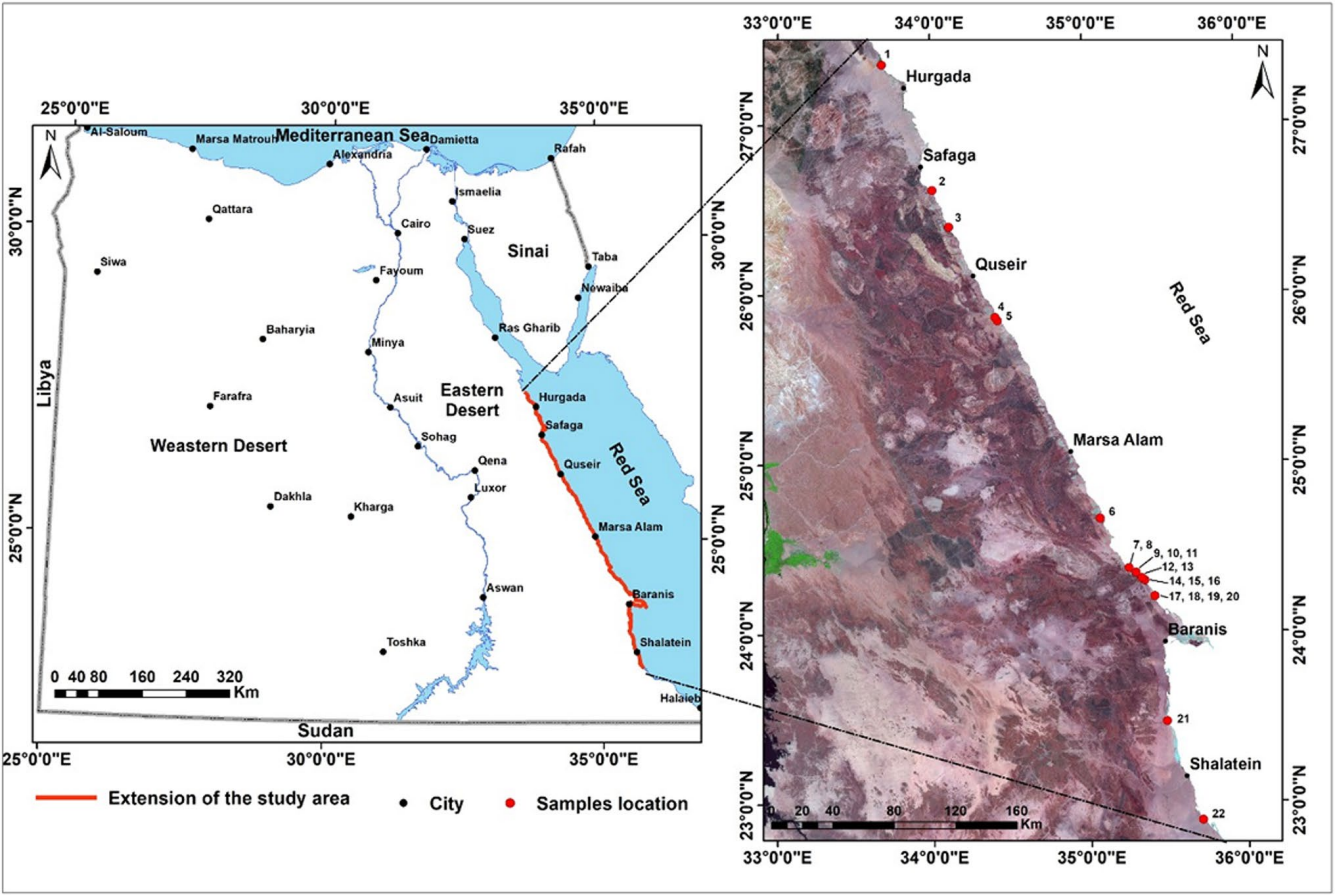
The location of the study area

The study area extends for about one thousand kilometers along the Egyptian Red Sea coast from north Hurghada city to south Shalateen city (Fig. 1). The location of 13 mangrove areas was identified, mapped, and validated from field visits. The study area mangrove stands scattered along the shoreline north from the Abu-Shaara site at Hurghada, south to Suez Gulf to the south of the Shalateen site at Halayeb province in the south border of Egypt with Sudan country. Most mangrove sites are covered by small patches or aggregations of stunted Avicennia *marina*, except in Shalateen site has two species of mangroves *Avicennia marina* and R. mucronate*.*

The area of study is located in an eastern desert condition with sparse rainfall and short showers with semi-periodic flooding on the Red Sea mountains drains towards the sea be loading by various metals (Azab 2009) to mangrove areas on the coast border.

### Sampling

Thirteen sites were visited and, in some sites, collected more than one sample according to the area of stand and, took two samples one towards the land and the other towards the sea. On the other hand, in some mangrove stands was difficult to collect two samples especially the sample towards the sea. So, the total number of samples was twenty-two (Table 1). The samples collected were from the surface sediment for inspecting the heavy elements. All samples were taken from 0.0 to 10 cm depth using a suitable grab sampler. The collected samples were put directly in air-sealed polyethylene bags and kept at 4 ℃ until analyses. Coordinates of sampling points were identified using a GPS instrument. The samples were air-dried (at room temperature) and the extraneous materials were removed.

**Table 1:** The name of the twenty-two site locations along the Red Sea coast

Sites	Name	Sites	Name
1	Abou-Shaara	12	Wadi EL Qulaan Delta2
2	K. 17 South Safaga	13	Wadi EL Qulaan Delta3
3	K. 35 North Qusier	14	Hamata1
4	Sharm El-Bahary	15	Hamata2
5	Sharm El-Qebly	16	Hamata3
6	Wadi EL Gemal	17	Wadi Lahmy1
7	Al Raada1	18	Wadi Lahmy2
8	Al Raada2	19	Wadi Lahmy3
9	Wadi Mastourah1	20	Wadi Lahmy4
10	Wadi Mastourah2	21	Marsa EL Hameera
11	Wadi EL Qulaan Delta1	22	Shalatein, Al-Somaa

### Chemical analysis for the heavy metals in the sediment

The sediment samples about (500 g) were desiccated in a hot air oven at 110 °C for 24 h, ground in a mortar, and then passed through a 2 mm plastic sieve. Well-mixed 2 g sediment samples were treated with 10 ml of freshly prepared aqua regia (HNO3 + 3HCl) on a sand bath for 2 h. After the samples were completely dried, the samples were dissolved in 10 ml of 2% HNO3, filtered via Whatman filter paper No 541, and then diluted to 50 ml with milli-Q water (Chen and Ma 2001). The acid-digested sediment samples were transferred into acid-washed plastic bottles and analyzed for to determine the concentration of Seven heavy elements (Mn, Ag, Cd, Cu, Pb, Ni, and Fe) using the ICP-MS analytical instrument in ppm.

### Indices of sediment contamination

Seven indices were utilized in this study to assess the contamination of the sediment as mentioned below:

#### Enrichment factor (EF)

Enrichment factor (EF) is used to differentiate metal origin from anthropogenic or natural sources. In the beginning, normalize the sample metal concentrations to reference elements, using iron in this study to determine whether a sediment sample is enriched with metals evaluated by the sample’s background environments. Equation (1) used to determine EF values, selected iron (Fe) as a normalizing element according to its major sorbent phase for trace metals and a quasi-conservative tracer of the natural metal-bearing phases in fluvial and coastal sediments (Schiff and Weisberg 1999; Turner and Millward 2000), expressed as

1 $${\text{EF}}=\frac{({\text{Cx}}/{\text{Fe}}){\text{sample}}}{({\text{Cx}}/{\text{Fe}}){\text{background}}}$$

where the Cx Sample and Cx background represent the concentration of selected metals. (Cx /Fe) background is the ratio of the background values of Fe. EF value of near unity means the elements that are naturally derived, while EF values of several orders indicate elements of anthropogenic origin. The classification of EF value according to Taylor (1964) to determine the degree of metal contamination is (i) EF < 2 = minimal, (ii) 2 ≤ EF < 5 = moderate, (iii) 5 ≤ EF < 20 = significant, (iv) 20 ≤ EF < 40 = very high, and (vii) EF ≥ 40 = extremely high.

#### Contamination factor (CF)

Contamination factor (CF) to assess the status of the surface sediment according to Hakanson (1980) based on the following equation:

2 $$\text{CF}=C\;\mathrm{metal}\;\mathrm{in}\;\mathrm{sediment}\;/\;C\;\mathrm{metal}\;\mathrm{background}$$

The CF values according to the four classes are depicted as follows:

(i) CF < 1 = low, (ii) 1 < CF < 3 = moderate, (iii) 3 < CF < 6 = considerable, and (iv) CF > 6 = very high.

#### Degree of contamination (Cd)

The degree of contamination (Cd) represents the sum of all the CF values for all the sampling sites. It was previously proposed by Hakanson (1980) as shown below:

3 $${\text{Cd}}={\sum }_{i=1}^{n}{\text{CF}}$$

The degree of contamination; (i) Cd < 6 = low, (ii) 6 < Cd < 12 = moderate, (iii) 12 < Cd < 24 = considerably high, and (iv) Cd > 24 = high.

#### Modified contamination degree (mCd)

Modified contamination degree (mCd) is the sum of all contamination factors for the element samples to the number of elements analyzed. This measure was proposed by Abrahim and Parker (2008) to investigate an unlimited number of heavy metals and is represented as

4 $${\text{mCd}}={\sum }_{i=1}^{n}{\text{CF}}/n$$

where *n* is the number of analyzed elements (i) is the element (or pollutant) examined and the contamination factor (CF).

#### Geo-accumulation index (Igeo)

Geo-accumulation index (Igeo) is used to analyze the level of pollution of trace elements and the contamination degree in marine sediments. It was initially described by Muller (1969) as Eq. (5):

5 $$\text{Igeo}=\text{log}2(\frac{\mathrm{Cn}}{\left(1.5\times\left(10\right)\mathrm{Bn}\right)})$$

where *Cn* is the trace metals calculated (measured concentrations of the sediment samples, respectively) and *Bn* is the background value (average value of crustal abundance) of a particular element.

Where; to decrease the possibility of variation in the background values for a specific trace element in the environment and minor anthropogenic influences, the concentration of each geochemical background value is multiplied by the factor of 1.5 Muller (1979). The sediment classification is based on the Igeo value as follows:

1. Igeo > 5 = extreme contamination,
2. 4–5 = strong to extreme contamination,
3. 3–4 = strong contamination,
4. 2–3 = moderate to strong contamination,
5. 1–2 = moderate contamination,
6. 0–1 = uncontaminated to moderate contamination,
7.  < 0 = uncontaminated.

#### Pollution load index (PLI)

Pollution load index (PLI) is a parameter to evaluate metal pollution in the marine environment, and it can be calculated from the following equation given by Tomlinson et al. (1980).

6 $$\mathrm{PLI}\;\mathrm{for}\;\mathrm a\;\mathrm{station}=\sqrt[n]{\text{CF}1\times\mathrm{CF}2\times\mathrm{CF}3..........\text{CFn}}$$

where CF is the contamination factor and n is the number of metals investigated.

A PLI value above one (> 1) indicates that an area is polluted, whereas values < 1 indicate no pollution or only background levels of pollutants are present (Chakravarty and Patgiri 2009; Mashiatullah, et al. 2013). While an estimation of PLI can be used to identify whether a site is collectively polluted or non-polluted by metals.

#### Potential ecological risk factor (E r) and risk index (RI)

The potential ecological risk factor is a method used to explore levels of contamination caused by metals and the risk to the aquatic environment. It was first introduced by Hakanson (1980). The formula is as follows:

7 $$Er=Ti\times Cf$$

where *Ti* is the toxic response factor and *CF* is the contamination factor.

The potential ecological risk index evaluates the environmental behavior and characteristics of heavy metal contaminants in the sediments. This method was previously proposed by Hakanson (1980) and its primary objective is to specify the agents that cause contamination. The RI is the summation of all risk factors for the detection of heavy metal contaminants in the sediment. The RI is calculated based on the following equation:

$${\text{RI}}={\sum }_{i=1}^{n}{\text{Er}}$$

Hakanson (1980) proposed a standardized toxic response factor of 1, 5, 5, 5, and 30 for Mn, Ni, Cu, Pb, and Cd.

### Multivariate statistical analysis

Descriptive statistics, correlations, and principal component analysis (PCA) are the most common multivariate statistical methods used in environmental studies (Ganugapenta et al. 2018; Islam et al. 2018; Saher and Siddiqui 2019). These methods were applied to verify significant relationships among the heavy metal’s sediments and to identify contamination sources (natural and/or anthropogenic). Principal component analysis has been applied to the data set of 22 sediment samples and seven variables (Cd, Cu, Pb, Ni, Ag, pb, and Fe). Pearson correlations analysis was applied (Berman 2016) to confirm the relations between different variables. R-mode factor analysis with VARIMAX rotation with the Kaiser–Meyer–Olkin (KMO) test with a > 0.5 KMO (0.5) (Hutcheson and Sofroniou 1999), as well as the Eigenvalues > 1, was applied to the measure’s metals in the sediment samples.

### Remote sensing and GIS analysis

#### Wadi basins extracting

The drainage system influencing the 13 mangrove sites was extracted using digital image processing of ASTER satellite data with a spatial resolution of 30 m utilizing the ArcGIS software environment by the “hydrology spatial analyst tool”; to extract basins in the study area. The Wadi’s have been selected according to every mangrove site, where there are sites that have received flooding from more than one Wadi. In addition to seawater current that transfers the drains load from north or south Wadi to the mangrove site. The satellite data was downloaded from the Copernicus Open Access Hub website https://scihub.copernicus.eu/.

The ancillary data of mineralization sites were obtained from the “Metallic and non-metallic deposits” maps with a scale of 1:1000000 that was issued by the Scientific Research Academy in 1998.

## Results and discussion

### Heavy metals in the sediments

All sample results for seven heavy metals, and the fundamental statistical (minimum, maximum, mean, and standard deviation) are shown in Table 2 to compare that with the concentration’s limits of the elements in the Earth’s crust after Turekian and Wedepohl (1961). The concentrations of Ag and Pb exceed the standard limits in all sites according to Turekian and Wedepohl (1961) metals limits. In contrast, Ni concentrations were high in stations; 1, 19, and 22 Abou-Shaar, Wadi Lahmy towards land and Marsa Sha’ab, respectively. The Cd was high at two stations 9 and 20 Wadi Mastourah and Wadi Lahmy, respectively.

**Table 2:** Heavy metal concentrations of sediment samples and limit values. Metal concentration (mg/kg dry weight)/(ppm) and their descriptive statistics

Sites	Mn [ppm]	Fe [ppm]	Ni [ppm]	Cu [ppm]	Cd [ppm]	Ag [ppm]	Pb [ppm]
1	107.518	3393.66	**151.968**	6.669	0.588	**0.839**	**81.143**
2	310.536	6151.02	50.519	7.297	0.779	**1.38**	**130.021**
3	281.892	5242.8	55.048	7.209	0.984	**1.309**	**88.424**
4	401.653	5023.01	63.435	7.085	0.651	**1.007**	**103.11**
5	152.167	3167.97	43.183	2.922	0.446	**0.996**	**60.046**
6	207.862	5228.3	29.897	11.413	0.903	**1.944**	**75.785**
7	661.333	23714.35	49.76	25.846	0.721	**2.722**	**188.137**
8	255.427	7856.06	28.844	19.805	1.394	**1.547**	**85.907**
9	210.346	4770.02	26.678	21.728	**2.557**	**1.46**	**83.687**
10	672.164	15013.65	43.657	21.328	1.212	**2.638**	**139.953**
11	525.113	21376.13	48.97	29.769	1.943	**6.095**	**140.105**
12	85.705	2332.33	22.206	8.618	1.222	**2.592**	**59.138**
13	175.563	3557.87	29.753	15.275	1.443	**4.307**	**68.688**
14	407.714	12849.09	37.45	31.824	1.688	**3.225**	**86.38**
15	733.352	23511.84	52.64	37.034	1.261	**3.883**	**188.334**
16	498.211	17652.62	37.477	17.754	0.751	**3.102**	**137.945**
17	371.433	17027.17	43.646	23.155	1.453	**2.547**	**120.983**
18	321.86	12596.37	49.311	23.807	1.036	**3.184**	**127.475**
19	635.897	20735.64	**124.515**	40.399	1.551	**2.742**	**120.525**
20	635.377	20586.83	**87.138**	42.218	**2.012**	**2.441**	**116.934**
21	363.249	10529.89	49.974	8.2	0.441	**1.111**	**90.941**
22	225.697	4372.23	93.549	13.904	0.504	**0.725**	**82.458**
Minimum	85.7	2332.3	22.2	2.9	0.4	0.7	59.1
Maximum	733.4	23714.4	152	42.2	2.6	6.1	188.3
Mean	374.5	11213.1	55.4	19.2	1.2	2.4	108
SD	198.3	7503.9	32.2	11.7	0.6	1.3	36.5
Reference	**850**	**57300**	**80**	**60**	**2**	**0.07**	**50**

Concentrations limits of the elements in the Earth’s crust after Turekian and Wedepohl (1961) and Alloway (1995)

B﻿oldface relate to the highest values

### Indices of sediment contamination

#### Enrichment factor (EF)

Enrichment factor analysis results for all samples are listed in Table 3. The values of EF related to the heavy metals noted Cu as minimal enrichment, whereas, Mn, Ni, Cd, and Pb showed significant enrichment. However, Ag showed as extremely high enrichment ranged from 202.37 to 1470.15 since it’s over 40 [ppm].

**Table 3:** Enrichment factor (EF)

Sites	Mn	Ni	Cu	Ag	Cd	Pb
1	2.135741	32.07365	1.876704	**202.3719**	3.309347	13.70053
2	6.168496	10.6623	1.132923	**332.8644**	4.384323	21.9533
3	5.599511	11.61817	1.313152	**315.7388**	5.538092	14.92988
4	7.978447	13.38829	1.347036	**242.8945**	3.66392	17.40953
5	3.02265	9.114002	0.880851	**240.2413**	2.510151	10.13842
6	4.128977	6.309921	2.084696	**468.9046**	5.082212	12.79586
7	13.13674	10.50211	1.040844	**656.563**	4.05789	31.76585
8	5.07381	6.08768	2.40754	**373.1458**	7.84563	14.5049
9	4.178319	5.630534	4.350137	**352.1609**	14.39116	14.13007
10	13.35189	9.214042	1.356648	**636.3016**	6.821308	23.63026
11	10.43086	10.33538	1.32996	**1470.151**	10.93548	23.65592
12	1.702447	4.686694	3.528742	**625.2062**	6.877589	9.985112
13	3.487389	6.279529	4.100101	**1038.875**	8.121409	11.59757
14	8.098843	7.904022	2.365297	**777.8896**	9.500304	14.58477
15	14.56733	11.10995	1.504241	**936.6032**	7.097087	31.79911
16	9.896478	7.90972	0.960485	**748.2213**	4.226735	23.29122
17	7.378156	9.211721	1.298691	**614.3519**	8.17769	20.42728
18	6.393437	10.40735	1.804939	**768.0002**	5.830755	21.52342
19	12.63148	26.27955	1.860615	**661.3871**	8.729248	20.34995
20	12.62115	18.39094	1.958446	**588.784**	11.32382	19.74363
21	7.215589	10.54728	0.743692	**267.98**	2.482011	15.35487
22	4.483252	19.74401	3.036967	**174.8744**	2.836584	13.92256
	EF < 2 Minimal	2 ≤ EF < 5 Moderate	5 ≤ EF < 20 Significant	20 ≤ EF < 40 Very high		EF ≥ 40 Extremely high

Boldface relate to the highest values

#### Contamination factor (CF)

The contamination factor analysis is shown in Table 4. An average CF value of Pb was moderately contaminated whereas Ag with an average of (33.63) indicates very high contamination sediment. The other metals Fe, Mn, Ni, Cu, and Cd are arranged as low contamination. The average CF results were ranked as the following: Fe < Cd < Mn < Cu < Ni < Pb < Ag.

**Table 4:** Contamination factor

Sites	Fe	Mn	Ni	Cu	Ag	Cd	Pb
1	0.05	0.13	1.90	0.22	**12.0**	0.20	0.81
2	0.09	0.37	0.63	0.24	**19.7**	0.26	1.30
3	0.08	0.33	0.69	0.24	**18.7**	0.33	0.88
4	0.08	0.47	0.79	0.24	**14.4**	0.22	1.03
5	0.05	0.18	0.54	0.10	**14.2**	0.15	0.60
6	0.08	0.24	0.37	0.38	**27.8**	0.30	0.76
7	0.36	0.78	0.62	0.86	**38.9**	0.24	1.88
8	0.12	0.30	0.36	0.66	**22.1**	0.46	0.86
9	0.07	0.25	0.33	0.72	**20.9**	0.85	0.84
10	0.23	0.79	0.55	0.71	**37.7**	0.40	1.40
11	0.33	0.62	0.61	0.99	**87.1**	0.65	1.40
12	0.04	0.10	0.28	0.29	**37.0**	0.41	0.59
13	0.05	0.21	0.37	0.51	**61.5**	0.48	0.69
14	0.20	0.48	0.47	1.06	**46.1**	0.56	0.86
15	0.36	0.86	0.66	1.23	**55.5**	0.42	1.88
16	0.27	0.59	0.47	0.59	**44.3**	0.25	1.38
17	0.26	0.44	0.55	0.77	**36.4**	0.48	1.21
18	0.19	0.38	0.62	0.79	**45.5**	0.35	1.27
19	0.32	0.75	1.56	1.35	**39.2**	0.52	1.21
20	0.32	0.75	1.09	1.41	**34.9**	0.67	1.17
21	0.16	0.43	0.62	0.27	**15.9**	0.15	0.91
22	0.07	0.27	1.17	0.46	**10.4**	0.17	0.82
CF < 1 = low		1 < CF < 3 = moderate		3 < CF < 6 = considerable		CF > 6 = very high	

Boldface relate to the highest values

#### Degree of contamination (Cd)

The degree of contamination was agreed from considerably high to very high in all sites as shown in Table 5 where the highest sample was 11 from EL-Qulaan mangrove sediments.

**Table 5:** The degree of contamination (Cd)

Sites	Value	Sites	Value
1	15.29	**12**	38.73
2	22.61	**13**	63.84
3	21.25	**14**	49.70
4	17.21	**15**	60.89
5	15.84	**16**	47.86
6	29.91	**17**	40.10
7	43.63	**18**	49.09
8	24.87	**19**	44.86
9	23.92	**20**	40.27
10	**41.77**	**21**	18.42
11	91.67	**22**	13.32
C d ≤ 6	**6 ≤ Cd ≤ 12**	**12 ≤ Cd ≤ 24**	**Cd ≥ 24**
Low	**Moderate**	**Considerable**	**Very high**

Boldface relate to the highest values

#### Modified degree of contamination (mCd)

Modified degree of contamination values for all sites as illustrated in Table 6 are categorized as moderate, high, and very high with a value of (13.10) at sample 11 from EL Qulaan mangrove sediments.

**Table 6:** Modified degree of contamination (m Cd)

Sites	Value		Sites	Value		
1	2.18		**12**	5.53		
2	3.23		**13**	9.12		
3	3.04		**14**	7.10		
4	2.46		**15**	8.70		
5	2.26		**16**	6.84		
6	4.27		**17**	5.73		
7	6.23		**18**	7.01		
8	3.55		**19**	6.41		
9	3.42		**20**	5.75		
10	5.97		**21**	2.63		
11	**13.10**		**22**	1.90		
mCd < 1.5	1.5 ≤ mCd < 26	2 ≤ mCd < 4	2 ≤ mCd < 4	8 ≤ mCd < 16	16 ≤ mCd < 32	mCd ≥ 32
Nil to very low	Low	Moderate	Moderate	Very high	Extremely high	Ultra-high

Boldface relate to the highest values

#### Geo-accumulation index

Geo-accumulation Index values of the heavy metals Table 7 noted that all sites were strong to extremely contaminated with silver while other sites were practically uncontaminated.

**Table 7:** Geo accumulation index (Igeo)

Sites	Fe	Mn	Ni	Cu	Ag	Cd	Pb
1	−4.663	−3.568	0.341	−2.754	**2.998**	−2.936	−0.886
2	−3.805	−2.038	−1.248	−2.625	**3.716**	−2.530	−0.206
3	−4.035	−2.177	−1.124	−2.642	**3.640**	−2.193	−0.762
4	−4.097	−1.666	−0.920	−2.667	**3.262**	−2.789	−0.541
5	−4.762	−3.067	−1.474	−3.945	**3.246**	−3.335	−1.321
6	−4.039	−2.617	−2.005	−1.979	**4.211**	−2.317	−0.985
7	−1.858	−0.947	−1.270	−0.800	**4.696**	−2.642	0.327
8	−3.452	−2.320	−2.057	−1.184	**3.881**	−1.691	−0.804
9	−4.171	−2.600	−2.169	−1.050	**3.798**	−0.815	−0.842
10	−2.517	−0.924	−1.459	−1.077	**4.651**	−1.893	−0.100
11	−2.007	−1.280	−1.293	−0.596	**5.859**	−1.212	−0.098
12	−5.204	−3.895	−2.434	−2.384	**4.626**	−1.881	−1.343
13	−4.594	−2.860	−2.012	−1.559	**5.358**	−1.641	−1.127
14	−2.742	−1.645	−1.680	−0.500	**4.941**	−1.415	−0.796
15	−1.870	−0.798	−1.189	−0.281	**5.209**	−1.835	0.328
16	−2.284	−1.356	−1.679	−1.342	**4.885**	−2.583	−0.121
17	−2.336	−1.779	−1.459	−0.959	**4.600**	−1.631	−0.310
18	−2.770	−1.986	−1.283	−0.919	**4.922**	−2.119	−0.235
19	−2.051	−1.004	0.053	−0.156	**4.707**	−1.537	−0.316
20	−2.062	−1.005	−0.462	−0.092	**4.539**	−1.161	−0.359
21	−3.029	−1.811	−1.264	−2.456	**3.403**	−3.351	−0.722
22	−4.297	−2.498	−0.359	−1.694	**2.788**	−3.158	−0.863
	Igeo ≤ 0	0 < Igeo < 1	1 < Igeo < 2	2 < Igeo < 3	3 < Igeo < 4	4 < Igeo < 5	Igeo ≥ 5
	practically	uncontaminated	moderately	moderately	strongly	strong	extremely

Boldface relate to the highest values

#### Pollution load index (PLI)

Pollution load index results clarify two pure sites (samples 5 and 12) Sharm El-Qebly and EL-Qulaan seaward sample. In contrast, finding 4 values with the level of deterioration according to PLI were 11, 15, 19, and 20 samples, from sites of EL-Qulaan and Hamata landward, whereas at the Lahmy site, the samples were from landward and seaward mangrove sediments. The other samples were ideal in the level of baseline represented in Table 8.

**Table 8:** Pollution load index (PLI)

Sites	Value	Sites	Value
1	0.001	**12**	0.000
2	0.005	**13**	0.006
3	0.003	**14**	0.151
4	0.003	**15**	1.590
5	0.000	**16**	0.096
6	0.003	**17**	0.147
7	0.382	**18**	0.103
8	0.110	**19**	1.744
9	0.009	**20**	1.418
10	0.215	**21**	0.004
11	1.392	**22**	0.002
	**0**	**1**	** > 1**
	Perfection	Baseline	Deterioration

Boldface relate to the highest values

#### Potential ecological risk factor

Potential ecological risk factors (Eri and RI) for sediments heavy metals are shown in Tables 9 and 10. All sites are represented low Ecological risk levels (Eri). As a result, the RI which is the summation of (Eri) confirmed a Low grade for all heavy metals excluding Cd with the value of (255.4) indicating a moderate potential ecological risk (Eri).

**Table 9:** Heavy metal contamination categories based on potential ecological risk

Sites	Mn	Ni	Cu	Cd	Pb
1	0.13	9.50	1.11	5.88	4.06
2	0.37	3.16	1.22	7.79	6.50
3	0.33	3.44	1.20	9.84	4.42
4	0.47	3.96	1.18	6.51	5.16
5	0.18	2.70	0.49	4.46	3.00
6	0.24	1.87	1.90	9.03	3.79
7	0.78	3.11	4.31	7.21	9.41
8	0.30	1.80	3.30	13.94	4.30
9	0.25	1.67	3.62	25.57	4.18
10	0.79	2.73	3.55	12.12	7.00
11	0.62	3.06	4.96	19.43	7.01
12	0.10	1.39	1.44	12.22	2.96
13	0.21	1.86	2.55	14.43	3.43
14	0.48	2.34	5.30	16.88	4.32
15	0.86	3.29	6.17	12.61	9.42
16	0.59	2.34	2.96	7.51	6.90
17	0.44	2.73	3.86	14.53	6.05
18	0.38	3.08	3.97	10.36	6.37
19	0.75	7.78	6.73	15.51	6.03
20	0.75	5.45	7.04	20.12	5.85
21	0.43	3.12	1.37	4.41	4.55
22	0.27	5.85	2.32	5.04	4.12
Average of (Eri)	0.44	3.46	3.21	11.61	5.40
RI (Sum of Eri)	9.69	76.23	70.54	255.40	118.81

**Table 10:** Classified levels of Eri and RI

Scope of potential ecological risk index (Eri)	Ecological risk level of single-factor pollution	Scope of potential toxicity index (RI)	General level of potential ecological risk
Eri < 40	Low	RI < 150	Low-grade
40 ≤ Eri < 80	Moderate	150 ≤ RI < 300	Moderate
80 ≤ Eri < 160	High	300 ≤ RI < 600	Severe
160 ≤ Eri < 320	Higher	600 ≤ RI	Serious
320 ≥ Eri	serious		

### Multivariate statistical analyses

Applying the Pearson correlations for the seven parameters revealed the high correlations between Fe with Mn, Cu, and Pb 0.9, 0.82, and 0.85, respectively. There is a significant correlation type moderate between Fe and Ag 0.6. On the other hand, a high positive correlation appeared between Cu, Mn 0.74, and a moderate correlation between Cu and Cd 0.65 (Table 11).

**Table 11:** Correlation matrix for the estimated parameters *n* = 7

Variables [ppm]	Mn	Fe	Ni	Cu	Cd	Ag	Pb
Mn	1.00						
Fe	0.91^**^	1.00					
Ni	0.12	0**.**10	1.00				
Cu	0.74**	0.82*	0.14	1.00			
Cd	0.21	0.29	−0.19	0.65	1.00		
Ag	0.45	0.60**	−0.26	0.59	0.49*	1.00	
Pb	0.85**	0.85**	0.05	0.56	0.05	0.44*	1.00

Bold characters indicate the significant correlations, usually greater than 0.6**, and values above 0.4*

Multivariate analysis (Principal component analysis, PCA) has been applied to the data set for 22 sediment samples and seven variables. Factor analysis is used to reduce the amount of data from 7 to 2 focusing on the most important variables that affect the study. This helps in interpreting the results and could build the appropriate model that represents the problem, in an inexpensive and objective description. The results give 4 factors. Eigenvalues above 1 are used to detect the most factors’ strength. Accordingly, two factors were extracted after the rotation for the data process.

Factor analysis can be summarized in (Table 12). The rotated component matrix of the factors was performed 0.7 for all 22 sites according to KMO. This also means the selected factor 1 and factor 2 comprise 70% of the total variance.

**Table 12:** Factor scores after Varimax rotation

Variables	F1	F2
Mn [ppm]	**0.95**	0.01
Fe [ppm]	**0.97**	0.13
Ni [ppm]	0.25	** −0.66**
Cu [ppm]	**0.82**	0.39
Cd [ppm]	0.24	**0.81**
Ag [ppm]	0.53	**0.65**
Pb [ppm]	**0.89**	** −**0.07
Variability (%)	52.80	23.98
Cumulative %	52.80	76.78

Rotated component matrix, extraction method: principal component analysis, rotation method: Varimax with Kaiser normalization

Boldface relate to the highest values

The first factor contributed 59.93% of the total Variance exhibiting high positive loadings on Fe and Mn. On the other hand, the second factor reflected 18.88% of the total variance and was composed of the following parameters Ag, Cd, Ni, Pb, and Cu, respectively.

### Remote sensing and GIS

**Fig. 2:**
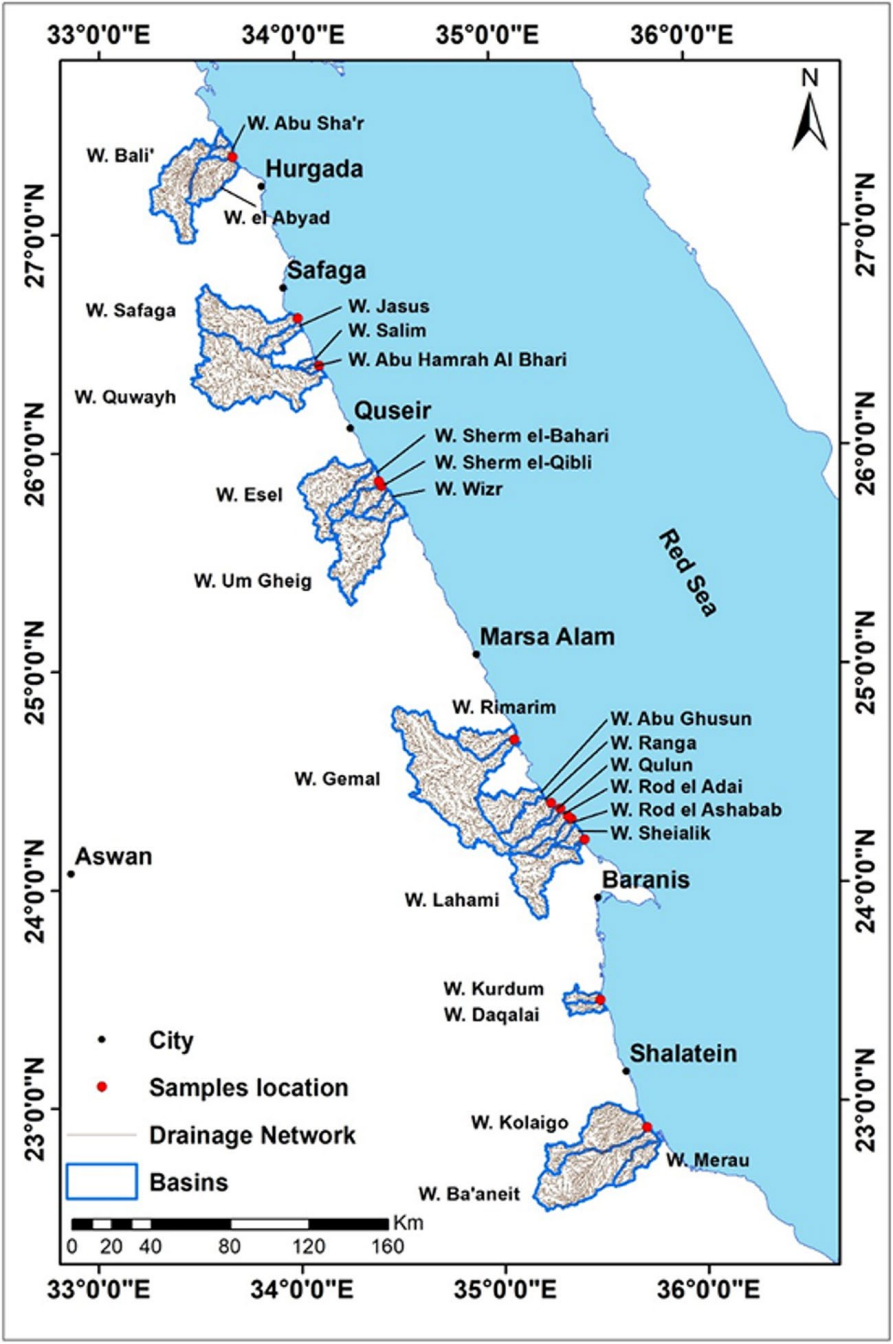
Drainage system of Wadis drains on mangrove sites

Remote sensing and GIS are utilized in this work to interpret the source of the trace element. The stream networks of the main Wadi were extracted and displayed the main mine sites of different elements. Thirteen mangrove sites along the coastline stand the termination of different Wadis flows from the Red Sea Mountains of the Eastern Desert are listed in Fig. 2 and Table 13. There are mangrove sites affected by more than one Wadi flow related to the coastal geomorphology and sea current flow where Wadis discharge the trace elements north or south. The high number of Wadis was in the south area from Wadi Qulaan to Wadi Lahmy reflected in the long coast with mangroves presence.

**Table 13:** The area of Wadis basins drained into mangrove sites

Name	Area KM^2^	Name	Area KM^2^
W. Bali’	779.66	W. Gemal	1967.54
W. Abu Sha’r	63.78	W. Abu Ghusun	395.95
W. El Abyad	386.06	W. Ranga	305.38
W. Safaga	747.78	W. Rod El Adai	218.80
W. Jasus	152.09	W. Qulaan	36.29
W. Salim	33.78	W. Rod El Ashabab	118.66
W. Abu Hamrah Al Bhari	28.16	W. Sheialik	80.53
W. Quwayh	1431.70	W. Lahami	595.02
W. Esel	674.75	W. Kurdum	90.28
W. Sherm El-Bahari	206.45	W. Daqalai	95.29
W. Sherm El-Qibli	160.19	W. Kolaigo	722.30
W. Wizr	114.62	W. Ba’aneit	999.84
W. Um Gheig	886.40	W. Merau	208.30
W. Rimarim	279.93		

**Fig. 3:**
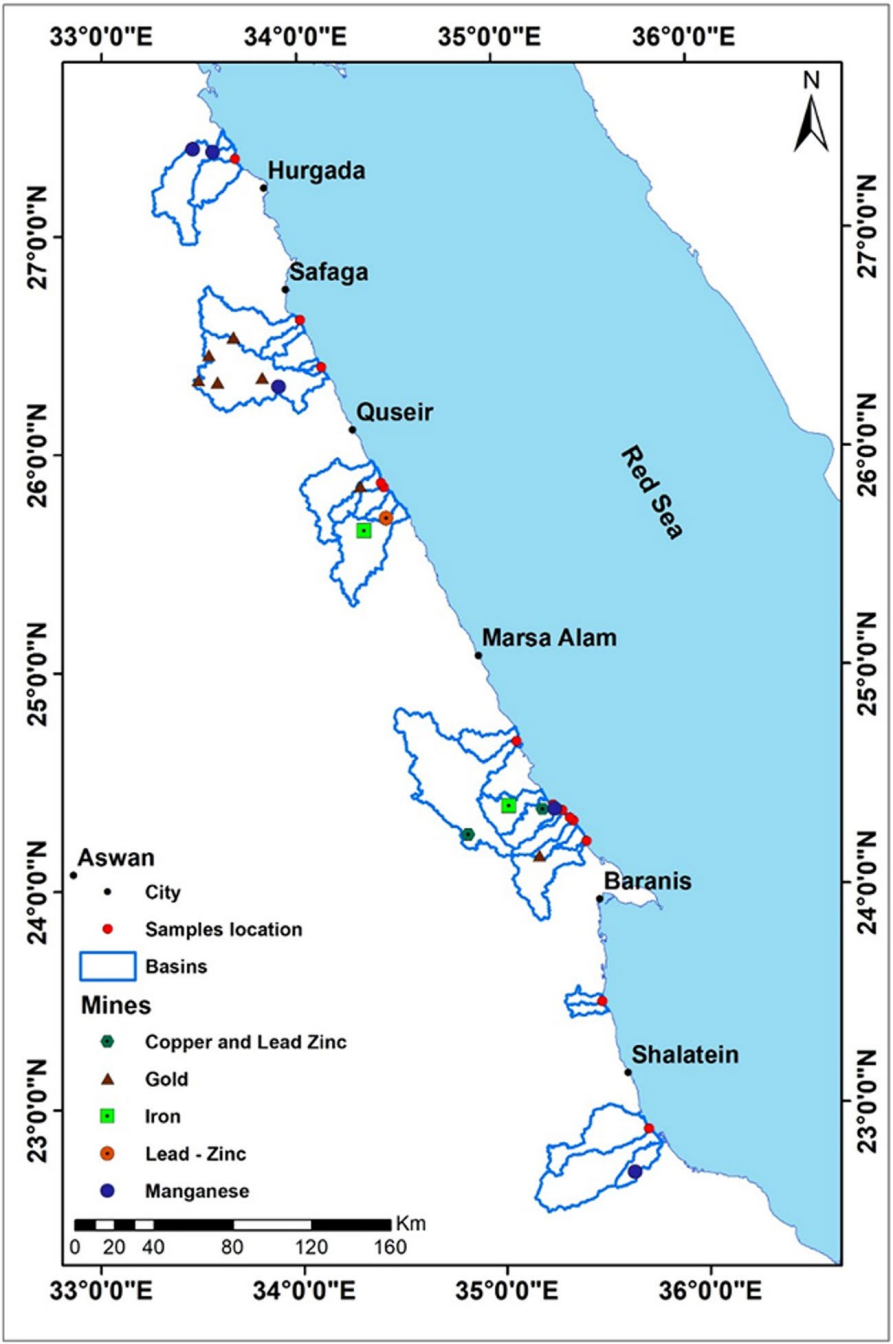
Drainage system and mining activity in the study area

According to the metallic and non-metallic deposit maps detecting the sites of 22 main mines at the Wadi’s basins of the study area as shown in Fig. 3 and Table 14. These mines are rich in different trace elements of copper, lead, zinc, iron, manganese, and gold. In addition, silver is detected with gold on the eastern desert rocks.

**Table 14:** The main mining locations in the study area

Code	Mines	Location	Longitude	Latitude
1	Manganese	Jabal Abu Shaar Qibli	33° 33′ 41.364″ E	27° 22′ 8.955″ N
2	Gold	Abu Muraywat	34° 17′ 39.845″ E	25° 50′ 14.991″ N
3	Gold	Simnah	34° 25′ 37.248″ E	25° 41′ 24.208″ N
4	Gold	Al Misikat—jabal Kabb Umayri	34° 19′ 3.251″ E	25° 38′ 4.582″ N
5	Gold	Jabal Kabb Umayri	34° 12′ 45.371″ E	25° 23′ 0.207″ N
6	Gold	Wadi Saqi	34° 34′ 58.006″ E	24° 48′ 53.606″ N
7	Manganese	Kalahin	34° 40′ 35.267″ E	24° 46′ 0.134″ N
8	Iron	Wadi Karim	35° 3′ 8.133″ E	24° 22′ 46.259″ N
9	Lead	Zuj Al Buhar- Isil	35° 13′ 1.082″ E	24° 21′ 53.607″ N
10	Gold	Sharm al Bahari	34° 50′ 47.074″ E	24° 15′ 8.131″ N
11	Lead—Zinc	Umm Ghayj	35° 12′ 21.500″ E	24° 9′ 14.447″ N
12	Iron	Umm Shaddad	33° 39′ 24.014″ E	26° 31′ 3.214″ N
13	Iron	Umm Nar	35° 3′ 23.236″ E	24° 7′ 24.909″ N
14	Gold	Jabal Al Hanjaliyah	35° 4′ 44.316″ E	23° 59′ 21.030″ N
15	Gold	Jabal Umm Ud	35° 38′ 12.058″ E	22° 41′ 48.080″ N
16	Iron	Abu Ghalqah	33° 32′ 14.743″ E	26° 26′ 12.762″ N
17	Copper and Lead Zinc	Hamata	33° 28′ 57.279″ E	26° 19′ 18.871″ N
18	Copper and Lead Zinc	Umm Samyuki	33° 34′ 53.433″ E	26° 18′ 48.998″ N
19	Gold	Abu Rahayah	33° 48′ 18.570″ E	26° 19′ 53.314″ N
20	Iron	Umm Ufayn	33° 53′ 10.989″ E	26° 17′ 38.275″ N
21	Copper and Lead Zinc	Jabal Darhib	34° 5′ 32.275″ E	25° 54′ 24.058″ N
22	Manganese	Jabal at Tiyur	34° 17′ 12.056″ E	25° 56′ 23.274″ N

## Discussion

The present study clearly showed spatial trends of heavy metal concentrations as the mean concentration of heavy metals was shown to decrease in the following order; Ag > Pb > Ni > Cu > Cd > Mn > Fe. The degree of pollution and health status of the sediment from seven geo-accumulation contamination factors analysis showed that the EF, CF, Cd, Igeo, Eri, RI, and PLI have fluctuated between the significant increases in the metal levels to moderate levels of ecological risk.

These results were in the same output for assessment of heavy metals contamination on mangrove sediments on the other side of the Red Sea, Suai Arabia that did not exceed the significant range from previous studies reported by Alzahrani et al. (2018); Alharbi et al. (2019); Aljahdali and Alhassan (2020); and Al-Hasawi (2022). Among the selected heavy metals, silver (Ag) concentrations were the highest according to the Igeo index which gives hints about the geographic sources. In addition to the presence of silver with gold in the most of region’s rocks (Zoheir and Lehmann 2011). The higher concentration of Ag in the sediments usually reaches the coastal mangroves drained from the Red Sea Mountains.

Principle component analysis has found the strength in the presence of Fe and Mn together is attributed to the environmental impacts and naturally occurred on mafic rock and granite respectively (Papachristou et al. 2014). Whereas, lead and Cadmium according to CF are moderately contaminated related to natural sources from Red Sea hills. Hanna (1992) noted that the total Cd content of the Red Sea sediment from 1934 and 1984 expeditions varied between 0.1 and 2 ppm, and he recommended that its origin is related to marine sediments lithogenous. Correspondingly, Mahdy et al. (2009) noted that lead–zinc mineralization is distributed between El Qusier and Ras Banas. In addition, cadmium is incorporated into the crystal structure of the zinc-lead minerals as a natural source. In the reverse Nour et al. (2022), that analysis heavy metals at Egyptian beaches on the Red Sea and the Gulf of Aqaba in Sinai and demonstrated that Cu, Pb, Cd, and Hg originated from anthropogenic sources. Moreover, Hanna (1992) pointed to the increasing concentrations of Ni, Pb, Cu, and Cd during the period (1934–1984) on the Red Sea coast. Where described the reasons from different sources of natural contamination as hot brine pools, and oil and minerals mining. In addition to anthropogenic impact the discharge of domestic, industrial waste, and marine transportation.

On the other hand, the PLI results suggest that the sediments are highly contaminated at Wadi EL-Qulaan, Hamata, and Wadi Lahmy as these are massive Wadis loading with elements discharge. Discussing the whole environment sediment metals sources that mangroves in the study area are situated in the coastal zone of the Eastern Desert of Egypt, which is dissected by the presence of rich alteration zones (El-Shafei 2011; Salem 2013; Amer et al. 2016; Ghoneim et al. 2018). These alteration zones are the perfect environment for several mineralization types including rare and heavy minerals. The mangrove sites are located on the end of Wadi’s that pass through the alteration zones. These Wadis act as the pathways/carriers of the washed minerals from the upstream rocks to the downstream outlet where the mangrove is present; thus, the carried minerals are objected to and deposited by the act of flooding the mangrove areas (Embabi 2018). The mining sites with several mineralization types fall within the drainage system and move with flooding to mangrove sites and are retained in sediment. This mineralization includes (copper, lead, zinc, gold, iron, and manganese) verified by Nour et al. (2019), Baioumy (2021), Abdelkareem and El-Shazly (2022), and Hegab et al. (2022). The existence of these mineralization sites within the stream network that ends up in the mangrove sites suggests the concentration of several heavy minerals being driven through the drainage network naturally.

The Red Sea mangrove in Egypt’s sediment is supposed to naturally originate from local minerals rather than contamination. In addition, it is located in protected areas with a conservation strategy (Okbah et al. 2005; El Daba and Abd El Wahab 2018) as the study results indicate weak enrichment. In contrast, other mangrove studies around the world showed that the increasing heavy metals contamination in mangrove sediment is due to pollution (Bodin et al. 2013; Rahman et al. 2014; Chai et al. 2019; Bakshi et al. 2019). In the case of the Mangrove, most of it is located in protected areas with conservation strategies.

## Conclusion

The heavy metals (Mn, Ni, Cu, Fe, Cd, Ag, and Pb) in the mangrove sediment are shown to be from natural origin, rather than anthropogenic activities. The remote sensing and GIS techniques successfully contributed to interpreting the pattern of the origin of heavy metals along the Red Sea coast. In addition, it represents a synoptic outline view of the study area with its relevant environmental condition and origin. Finally, the investigation discovered a high content of silver metal concentrated in mangrove sediments, which necessitates rigorous further research to understand how to apply sustainable exploitation of this metal’s economics to ignore the negative impact on marine ecosystems.

## Data Availability

All included in the manuscript.
